# Histopathological and radiographic characterization of the lesions of pododermatitis in sheep: support for the establishment of the foot injuries degree and its prognosis

**DOI:** 10.3389/fvets.2025.1567665

**Published:** 2025-04-25

**Authors:** Caroline da Silva Silveira, Raissa Moreira de Morais, Pedro Araújo Damboriarena, Ricardo Pozzobon, Martín Fraga, Bruno Leite dos Anjos

**Affiliations:** ^1^Plataforma de Investigación en Salud Animal, Instituto Nacional de Investigación Agropecuaria (INIA), Estación Experimental La Estanzuela, Colonia, Uruguay; ^2^Programa de Pós-graduação em Ciência Animal, Universidade Federal do Pampa, Uruguaiana, Rio Grande do Sul, Brazil; ^3^Laboratório de Patologia Veterinária, Universidade Federal do Pampa, Uruguaiana, Rio Grande do Sul, Brazil

**Keywords:** lameness, foot disease, diagnosis, ovine, pathology

## Abstract

Foot diseases in small ruminants cause locomotor disorders, leading to significant economic, productive, and health concerns in sheep and goat farming worldwide. The diagnosis and classification of lesions caused by footpad dermatitis are complex and based only on clinical observations in the field. In this context, this study assessed the histopathological and radiographic characteristics of lesions caused by pododermatitis in sheep to improve and deepen the classification of lesions and optimize diagnosis and prognosis. In this study 1.701 lame sheep were included and were distributed across 21 farms in southern Brazil. Lesions were categorized into three severity grades based on clinical, histological and radiographic observations. As a result of these findings, the lesions were categorized into 3 grades described as: mild interdigital dermatitis (grade 1), necrosis with bone involvement (grade 2) and severe tissue loss with osteolysis (grade 3). Radiographic evaluation revealed bone changes ranging from mild inflammation to osteomyelitis and pathological fractures in advanced grades. Histologically, in most severe cases, progressive inflammation, thrombosis and necrosis were observed. The results suggest that regardless of the origin of pododermatitis, whether related to environmental factors and/or agents such as *Dichelobacter nodosus* and *Fusobacterium necrophorum*, the lesions are progressive and severe. This adapted classification system can help field technicians and producers to effectively diagnose and treat these lesions depending on the grade, limiting their progression and consequently reducing economic losses. This integrated approach can improve animal welfare and productivity in South American herds, where these diseases are a significant concern.

## Introduction

Foot diseases are important injuries that cause locomotor disorders in small ruminants (1). In most countries where sheep and goat farming is of sociocultural and economic importance, diseases that cause lameness are a cause of concern, as they cause severe economic, productive, and reproductive losses and compromise animal health and well-being (2–5).

Infectious pododermatitis, also known as footrot and interdigital dermatitis, are infectious and contagious foot diseases that are prevalent in sheep flocks around the world (1, 2, 6–8). Although these diseases account for more than 90% of the cause of lameness in sheep in the United Kingdom (9) more recently, another disease known as contagious ovine digital dermatitis (CODD) has been described, which raises additional concerns regarding the health of livestock in this country (10–14).

Studies quantifying the economic losses generated by foot diseases are scarce. It is estimated that between treatment, loss of animal performance, and preventive measures in sheep with lameness, cost to the UK industry around £24–80 million per year (15, 16). In Australia, footrot is the main cause of hoof disease in sheep with an impact of over 44 million Australian dollars per year to the meat and wool industries (17). However, in South America, there is no literature available on the quantification of economic losses caused by foot diseases in small ruminants.

At the flock level, footrot can also be classified as benign, intermediate, and malignant; this classification is based on the clinical aspect and flock affection measures (18).

Although pododermatitis are widely present in ovine, and producers and technicians are aware of their presence, it is still difficult to assign lesions to each disease and to characterize the severity of damage in sheep’s hooves (19, 20). The different stages of treatment are progressive and associated with environmental and management factors, such as vaccination and hoof cutting, and a more specific and targeted clinical approach is extremely necessary for a more successful diagnosis and prognosis (13, 21, 22).

The diagnosis of foot diseases is generally based on clinical inspection and macroscopic characteristics of the lesions. The classification system proposed in the 1970s is still widely applied worldwide and is based on scoring lesions according to the damage (23). This system classifies lesions from 0 for healthy hooves to 4 for a severe lesion (23). Although this system, which describes only clinical lesions, has been adapted and modified for the differential diagnosis between pododermatitis such as footrot (12, 24–26) and CODD (12) over the years.

In this context, the correct treatment and control of diseases can be carried out depending on the precise diagnosis of the cause. To facilitate the clinical diagnosis and assist in the control of ovine farms, this work aims to adapt and deepen the pre-existing classification system for foot lesions in sheep, also considers the histological and radiographic aspects.

## Materials and methods

### Study strategy and sampling

Twenty-one sheep farms, located in the Southwest Mesoregion of the state of Rio Grande do Sul, Brazil, were included through a non-probabilistic observational study. These farms were previously characterized (21), and additional details on the origin, total number of animals on the farms, number of sick animals, severity of foot lesion, and treatment used are presented in Supplementary material.

Between April 2014 and April 2015, veterinarians at these farms identified a total of 1,701 sheep with lameness due to foot lesions. These animals were segregated, and a detailed macroscopic inspection and photographic record of all four hooves (diseased and healthy) were made. When the death of these animals occurred due to culling or other reason, every hoof was collected and preserved in formalin until shipment to the laboratory installations.

After photographic recording, the macroscopic lesions were analyzed by the research team and morphologically characterized according to the severity of the lesions in 3 grades (Table 1) adapting the Egerton and Roberts classification system (23) and the histological and radiographic lesions were evaluated as explained below.

**Table 1:** Classification of pododermatitis grades through macroscopic interpretation.

Classification	Macroscopic interpretation
Grade 1	Mild interdigital dermatitis
Grade 2	Necrotizing dermatitis with hoof involvement
Grade 3	Severe lesions with deformity and tissue loss

Adapted from Egerton and Roberts (23).

#### Radiographic evaluation

Formalin-preserved hooves were processed at the Hospital Universitário Veterinário of the Universidade Federal do Pampa, Brazil. The limbs were removed by radiocarpal or tibiotarsal disarticulation, then gently washed with running water to remove organic matter. Nineteen limbs of sheep with macroscopic lesions were evaluated, and 2 healthy limbs were selected as controls.

The radiological examination was performed in four projections: lateromedial, dorsopalmar, and two oblique projections (dorsolateral-palmaromedial and dorsomedial-palmarolateral). The radiographs were performed with a Phillips Áquilla Plus 300 devices, using a regime of 45 KV and 15 mAs. Subsequently, the images were edited in the Kodak DirectView EPV software and converted to JPG format. The radiographic findings were classified according to the degree of bone involvement of the phalanges and the extent of the lesions.

#### Histological evaluation

**Figure 1:**
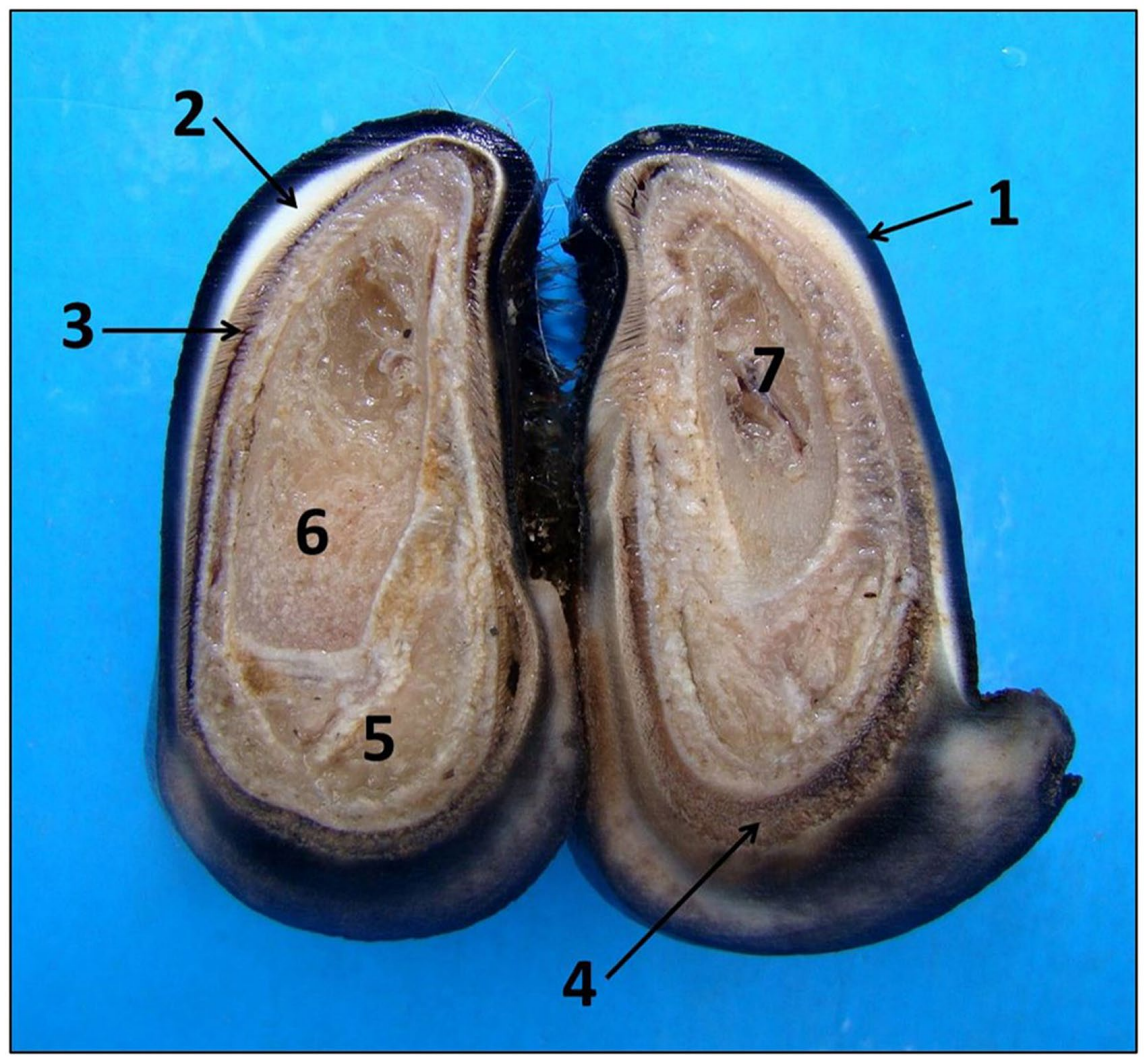
Anatomy of sheep’s hooves seen in cross section. Observed macroscopically from the outside to the inside: wall with external strata (1), middle (2) and laminar (3), coronary region (4), chorion (5), distal phalanx (6), and bone marrow (7).

For histological evaluation, formalinized hoof and skin samples were processed. Each limb was cut with a hand saw on both digits at the same time in a cut perpendicular to the dorsal surface of the hoof, obtaining two transversal fragments with a thickness of 0.3 cm. This sample was collected 0.2 cm after the hoof crown. Upon cutting, the following structures were observed: external stratum, middle stratum, laminar stratum, and distal phalanx (Figure 1). From this, the laminar stratum was identified and separated from the hard keratin of the hoof wall with the aid of a scalpel.

The skin fragments were removed from the interdigital region in a cut of approximately 0.1 cm that extended the dorsopalmar or plantar from the epidermis to as close as possible to the periosteum of the phalanges and the dorsal region of the limb.

**Figure 2:**
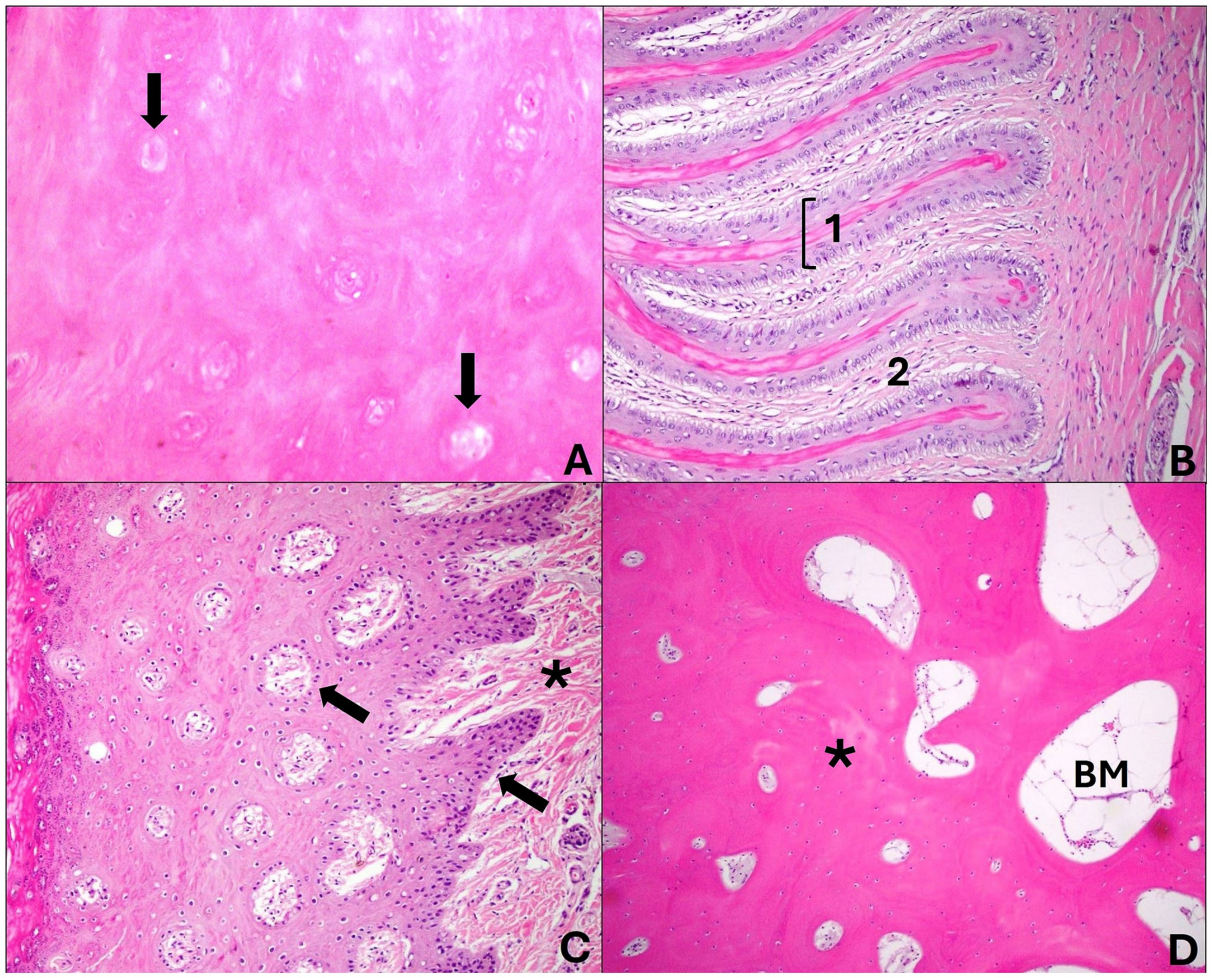
Cross sections of a healthy sheep’s hoof. **(A)** Middle layer: abundant keratinized cellular matrix amid the tubules formed by keratinocytes located around a hollow, unpigmented central medulla (arrows). Hematoxylin and eosin stain, 40×. **(B)** Laminar layer: presents epidermal layers (1) that join the dermal layers (2) in this region and are arranged parallel to each other. Note that the laminar chorion merges with this structure. Hematoxylin and eosin stain, 20×. **(C)** Coronary region—the coronary epidermis presents dermal papillae (arrows) that join the coronary chorion (*); there is a thick stratum spinosum and a thin stratum granulosum and corneum. Hematoxylin and eosin stain, 20×. **(D)** Distal phalanx: the bone forms mature bone trabeculae (*) in the middle of the bone marrow (BM). Hematoxylin and eosin stain, 20×.

The bone fragments were subjected to slow decalcification using formic acid for 7 days to soften the distal phalanx and then processed. All fragments (hoof and skin) were routinely processed for histology, stained with hematoxylin and eosin (HE) and analyzed under an optical microscope. For this evaluation, 36 injured digits and 4 digits from non-injured hooves were used as control (Figure 2).

Grading of histological lesions was performed in a blind study. The type and time of occurrence of the lesion were considered, as well as the distribution and intensity of the morphological changes.

## Results

### Lesion grading system

#### Grade 1

**Figure 3:**
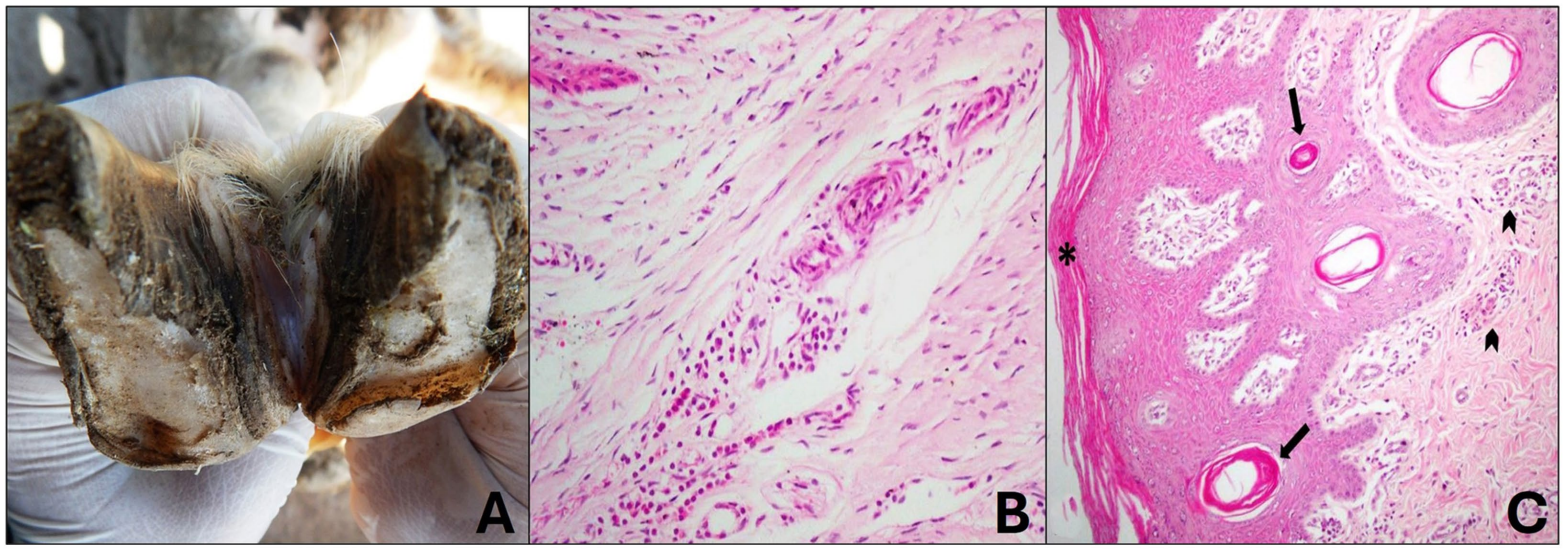
Grade 1 ovine pododermatitis lesion. **(A)** Sheep hooves. Slight lesion in the interdigital space with focal alopecia is observed. **(B)** Laminar chorion with mild perivascular lymphoplasmacytic inflammatory infiltrate is observed. Hematoxylin and eosin stain, 20×. **(C)** In the interdigital skin, there is mild orthokeratotic hyperkeratosis (*) and mild multifocal infundibular hyperkeratosis (arrow); mild diffuse lymphoplasmacytic dermatitis is also noted in the superficial dermis (arrowhead). Hematoxylin and eosin stain, 10×.

Sheep that macroscopically presented focal and mild lesions of the interdigital skin were classified as grade 1. These lesions were characterized by redness and alopecia of the interdigital skin (Figure 3A), but no hoof wall involvement was observed at this stage. No bone changes were observed radiographically.

Histologically, the changes were restricted to the laminar chorion and interdigital skin and were characterized by mild perivascular lymphoplasmacytic coronitis (Figure 3B). Mild orthokeratotic hyperkeratosis was observed in the epidermis of the interdigital skin and, in some cases, mild cytoplasmic vacuolization of keratinocytes. The dermis showed mild multifocal infundibular hyperkeratosis, mild diffuse hyperemia, and mild lymphoplasmacytic inflammatory infiltrate scattered throughout the superficial dermis (Figure 3C).

#### Grade 2

**Figure 4:**
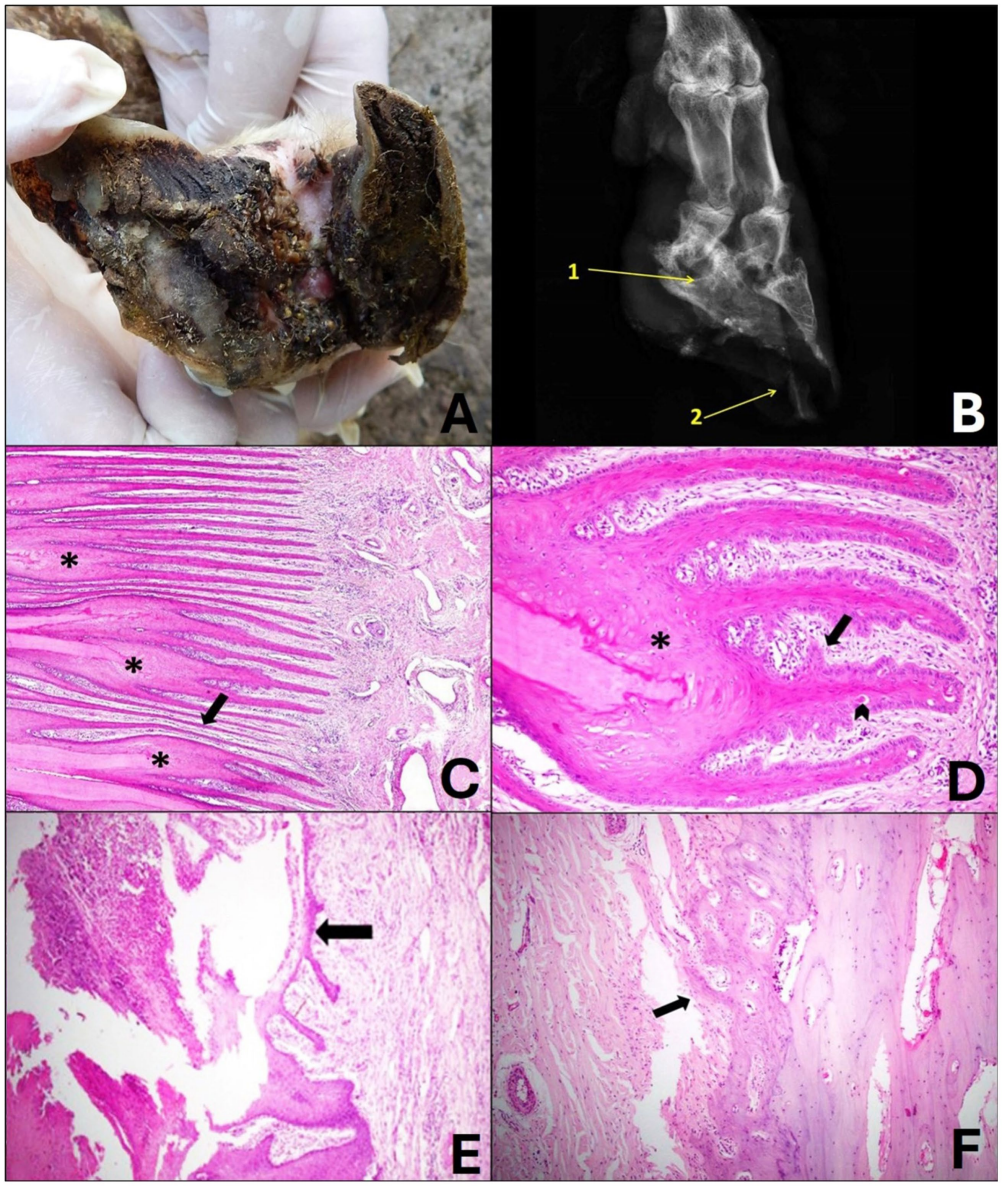
Grade 2 ovine pododermatitis lesion. **(A)** Solar surface of the sheep hoof. Note the accumulation of organic matter in the middle of the lesion. In the axial region, moderate detachment of the wall is observed, extending toward the dorsal roof; dermatitis of the interdigital skin is also noted. **(B)** Radiographic image in dorsolateral-palmaromedial oblique projection. Bone sclerosis and radiolucent area (lysis) of the distal interphalangeal joint (1), bone remodeling of the third phalanx, irregularity of the periosteum of third and second phalanx and area of destruction of the horny sheath marked by a radiolucent region in the hoof pincer (2) can be observed. **(C)** It can be observed that the epidermal laminae of the laminar stratum are asymmetric and markedly thin (arrow), there is fusion and shortening (*), and it is still possible to visualize moderate diffuse lymphoplasmacytic coronitis. Hematoxylin and eosin stain, 10×. **(D)** The laminae are fused and shortened (*), in their structure the papillae project from the basal lamina toward the papillary chorion and are merging with each other (arrow). Note dyskeratosis along the laminae (arrowhead). Hematoxylin and eosin stain, 20×. **(E)** In the region of the coronary ulcer, focally extensive crusts, fibrin and marked neutrophilic infiltrate are observed; a thin layer of re-epithelialization of the epidermis is also noted (arrow). Hematoxylin and eosin stain, 10×. **(F)** In the bone of the 3rd phalanx, the lesion was characterized by moderate irregularity of the periosteal surface that projects toward the coronary chorion (arrow). Hematoxylin and eosin stain, 10×.

In sheep that were classified as grade 2, the lesions were moderate to severe and had a focally extensive distribution in the interdigital and dorsal skin, abaxial and axial regions of the hoof wall. Deep interdigital dermatitis with ulcers and pus, edema and alopecia, focal necrosis of the periople and axial region were observed (Figure 4A), sometimes the lesions were aggravated by parasitism with compatible larvae of *Cochliomyia hominivorax*. These changes extended from the palmar/plantar surface to the dorsal surface, and necrosis also occurred in the axial region of the hoof wall, which advanced toward the abaxial region of the digit. In this grade, it was observed that in some cases the hoof wall began to detach from the soft tissues and veterinarians reported a foul odor.

Radiographically, there was slight bone sclerosis, irregularity of the periosteum interpreted as osteitis, and slight bone remodeling of the third phalanges (Figure 4B).

Histologically, the digit showed involvement of the laminar layer, chorion, coronary region, bone and interdigital and dorsal skin. In addition, it was possible to observe papillae that originated from the basal lamina toward the laminar chorion with subsequent fusion, as well as abrupt and individual keratinization of the keratinocytes. The epidermal layers showed marked lesions such as asymmetry, shortening and fusion (Figures 4C,D). In some cases, the coronary region presented areas that varied from focal necrosis of keratinocytes to focally extensive areas with intense neutrophilic inflammatory infiltrate that extended close to the periosteum of the third phalanx and myriads of bacteria amidst fibrin and crusts (ulcers) (Figure 4E).

Congestion and mild hyperemia with dilated blood vessels, diffuse lymphoplasmacytic coronitis and some Mott cells were also noted in the chorion. At this level, the third phalanx presented moderate periosteal reaction, and a focus of irregularity was observed on the surface of the periosteum that sometimes proliferated toward the chorion (Figure 4F).

In the interdigital and dorsal skin, the lesions were quite similar, characterized by moderate ortho-keratotic hyperkeratosis, focal ulcer, moderate to severe diffuse lymphoplasmacytic inflammatory infiltrate, and in some cases moderate congestion and hyperemia.

In the deeper dermis of the digits of some sheep, a focally extensive area of eosinophilic infiltrate surrounded by connective tissue and epithelioid macrophages (granuloma) was noted.

#### Grade 3

**Figure 5:**
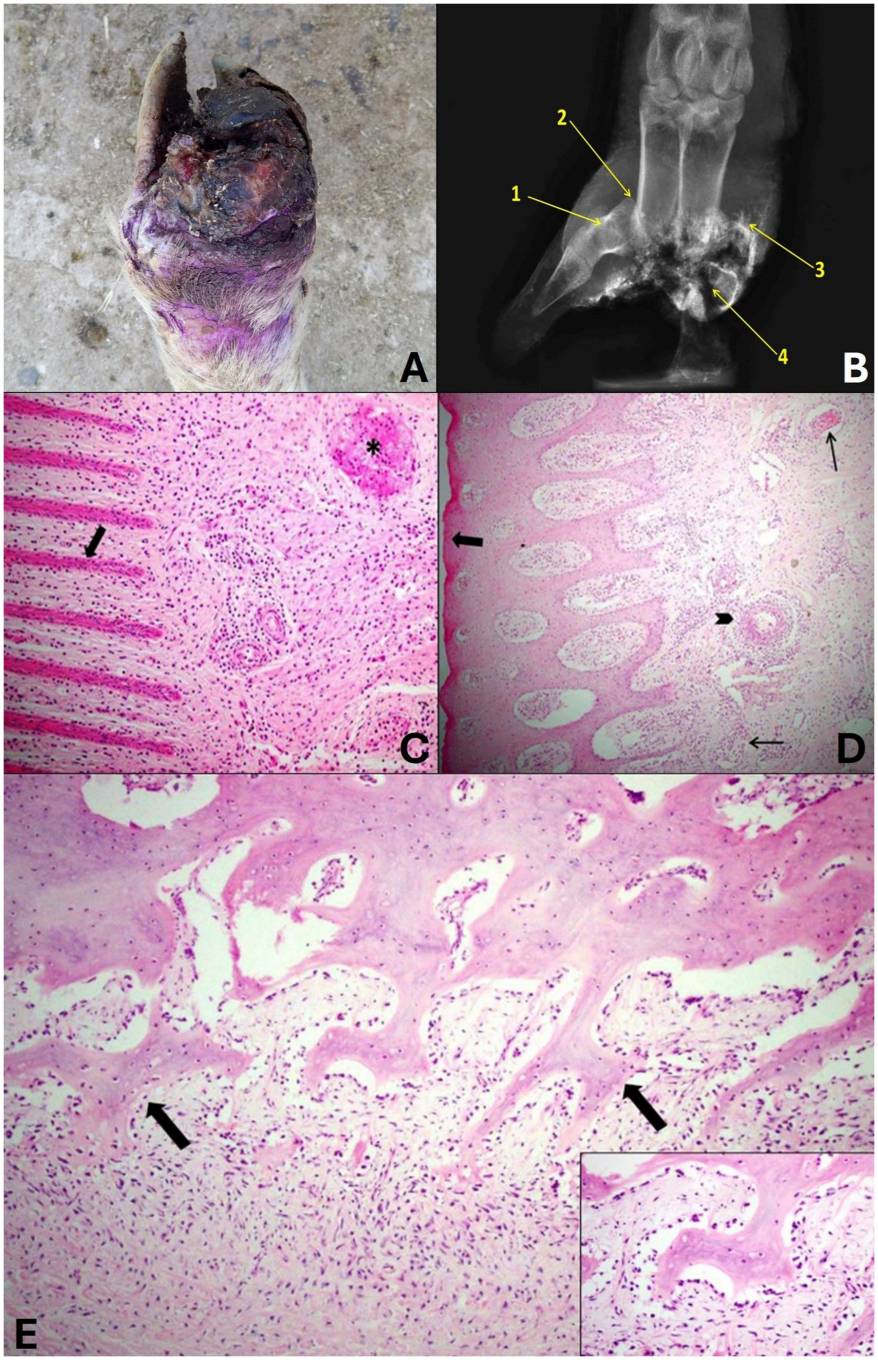
Grade 3 ovine pododermatitis lesion. **(A)** Dorsal surface of the distal region of the limbs of sheep. Note the severe lesion with marked tissue loss and deformity of the hoof digits, edema with increased volume in the distal portion, redness, detachment of the hoof wall and proliferation of granulation tissue in the region of the coronary band and interdigital region. **(B)** Radiographic image in dorsolateral-palmaromedial oblique projection. Osteitis of the middle phalanx (1), dislocation of the proximal interphalangeal joint (2), exostosis (3) and bone fragments of the proximal, middle and distal phalanges and the sesamoid (4) can be observed. **(C)** In the laminar layer, the epidermal papillae are markedly thin and shortened (arrow). In the chorion, there is a marked multifocal mixed inflammatory infiltrate and thrombi (*). Hematoxylin and eosin stain, 20×. **(D)** In the coronary region, the granular layer is absent (arrow), the blood vessels are dilated and filled with neutrophils (thin arrow), and there is neutrophilic arteritis (arrowhead). Hematoxylin and eosin stain, 10×. **(E)** In the bone of the 3rd phalanx, there is marked proliferation and irregularity of the periosteum that projects toward the chorion (arrows). It also presents lymphoplasmacytic and neutrophilic inflammatory infiltrate around the periosteum (periosteitis). Hematoxylin and eosin stain, 20×. In detail, numerous active osteoblasts can be seen surrounding the bone trabeculae. Hematoxylin and eosin stain, 40×.

In grade 3, changes were observed in the foot of the sheep that were classified as severe with tissue loss. The digits of these sheep presented lesions in the interdigital skin that extended to the coronary band, hoof wall and third phalanx in more severe cases. In addition to marked parasitism by compatible larvae of *C. hominivorax*. Marked swelling of the distal region of the limb and alopecia could also be observed. In this grade, the hoof presented total detachment of the horny sheath (Figure 5A). It was also observed that one or both distal phalanges were exposed and fragmented or were not observed due to the marked bone lysis. In all cases, a marked putrid odor was reported.

When the phalanges were present, the radiographic lesions were characterized as severe, with osteomyelitis in practically all phalanges, characterized by osteolysis of the region, sometimes total lysis of the distal and middle phalanges, exostosis and pathological fractures. Only osteitis was observed in some middle and distal phalanges, in addition to osteolysis of sesamoids and sometimes phalangeal dislocations due to joint infection and anatomical changes resulting from foot lesions (Figure 5B).

Histologically, marked changes were observed in all structures that make up the hoof, when these were present. In the laminar layer, it was noted that the epidermal papillae were markedly thin and shortened. In the coronary region, there was an absence of the granular layer, and the blood vessels of the dermal papillae were dilated and full of neutrophils. In the chorion, a marked multifocal mixed inflammatory infiltrate was observed and was characterized by lymphocytes, plasma cells, macrophages and neutrophils, there were dilated vessels full of neutrophils, neutrophilic arteritis and thrombi were also observed (Figures 5C,D). In the bone, it was possible to observe a marked periosteal reaction and foci of irregularity on the surface of the periosteum that proliferated toward the chorion. In addition, there was lymphoplasmacytic and neutrophilic inflammatory infiltrate in the periosteum (periostitis) and active osteoblasts (Figure 5E). It was not possible to evaluate the skin due to the absence of tissue in the regions proposed in the study.

## Discussion

This work was based on the morphological evaluation of the various patterns of foot lesions in sheep (1, 12, 14, 23, 24, 26–28). Based on this clinical evaluation, this article provides, at least in South America, the first standardized and detailed description of the macroscopic, radiographic, and histological presentations of sheep hooves affected by pododermatitis.

It was possible to classify and visually record the lesions into three degrees of severity, which varied from 1 (discrete focal lesions in the interdigital skin) to 3 (severe lesions with tissue loss). Through this, technicians and sheep farmers will be able to systematically and consistently identify the problem in their flocks. However, according to Kaler and Green (9) the experience of technicians and producers in naming and recognizing foot lesions is still a challenge for field diagnosis, in which the majority wrongly attribute or name any hoof lesion as footrot. Possibly, this wrong recognition was formed by the perception and experience of the disease previously occurring at the herds and is independent of the classifications and technical nomenclatures of the lesions.

Within the samples analyzed, the findings described helped to define the extent of the lesion and allowed visualization of the intensity of bone and adjacent soft tissue involvement in different degrees. These results may explain, in part, why many animals continue to lame after the lesions heal. Significant radiographic abnormalities, including soft tissue and bone alterations, were evident in digits submitted to a five-point lesion classification system in sheep affected by CODD. In addition to the marked pathological changes, radiographic evaluation revealed an association between increased locomotion score and worsening of the disease degree (12).

Effective treatment and prevention criteria are essential for the welfare and profitability of the sheep flock (29). Total costs per sheep/year per flock were used to investigate the relative cost–benefit of different methods of controlling lameness. Total costs were significantly lower in flocks where sheep farmers followed the best practices to minimize the prevalence of lameness in sheep, including prompt treatment with parenteral and topical antibiotics, and avoiding whole-flock hoof trimming and routine footbaths (30). The foot injury classification system proposed in this study may assist in treatment and control strategies even before disease progression leads to severe and potentially irreversible lesions.

The hooves classified as grade 1 were characterized by mild interdigital dermatitis and interdigital alopecia, but without involvement of the hoof wall. The lesions described in the study classification are compatible with infection caused by *F. necrophorum* (interdigital dermatitis) and non-virulent strains of *D. nosodus* and correspond to what is described as benign footrot (1, 5, 18, 24, 31, 32). Regardless of the cause and/or etiological agent, the lesions observed in this category can be treated with the use of foot baths, as they are superficial changes in the interdigit in which the disinfectant (formalin, zinc sulfate) can act effectively.

Even though it is suggested to performed microbiological and molecular evaluation for the differential diagnosis between interdigital dermatitis and footrot, the interpretation of the results can be confusing. Moore et al. (33) state that establishing the correct diagnosis of the disease is complex, since *D. nodosus* is present and has been isolated from numerous digits diagnosed as interdigital dermatitis. In the case of interdigital dermatitis or a milder form of footrot, macroscopic lesions observed in the field should not be neglected. They should be treated, since once contaminated with *D. nodosus*, the progression of the lesion is inevitable.

Grades 2 and 3 were classified by the severity of the lesions, characterized by the presence of necrosis and severe involvement of the digit with pedal osteitis, and in some cases, tissue loss with bone lysis. Although the macroscopic lesions described in this study are classic of the virulent form of footrot (21, 23–27), the grades were better characterized and the lesions observed clinically are complemented/supported by radiographic and histological interpretation.

Radiographic evaluation confirmed that sheep with grades 2 and 3 lesions, presented bone inflammation and, in more severe cases, bone loss, which increases the chance of these animals progressing to tissue loss with consequent lameness and relapses. These changes are caused by the devitalization of the interdigital skin due to susceptible factors, such as local humidity and heat, and the facilitation of infection by the highly virulent *D. nodosus*, a pathogen that destroys the hoof wall (34).

Histologically, it was noted that in grades 2 and 3 there is moderate to severe laminitis that progresses to detachment of the hoof wall. This might be due to the marked inflammatory infiltrate and thrombi that triggers gangrene of the distal portion of these limbs, explaining the serious injuries that often do not respond to the treatments applied. Angell et al. (28) also noted that the histopathological evaluation of cases of sheep with COOD was fundamental to clarify therapeutic approaches.

The studies that characterize pododermatitis based on clinical features and lesion morphology are carried out in other environmental and sheep farming conditions and do not entirely converge in several aspects of their concepts with what occurs in South America. This makes it difficult for technicians and producers in the region to classify and recognize lesions and consequently potential treatment.

In addition to being a disease that causes productivity and economic losses (35), secondary complications due to extensive parasitosis caused by *C. hominivorax* larvae were observed. Myiasis is one of the main problems in animal and public health in South America due to aggravation of animal lesions mainly in extensively raised livestock (36). In this work this parasitosis was considered as one of the factors to be taken into consideration when grading into 2 or 3.

As observed in this study, it is believed that most foot injuries presented by sheep indicate different stages of evolution. To facilitate the conceptual understanding of this condition in sheep, we suggest that it is a polyphasic and multifactorial condition, caused by *D. nodosus*, *F. necroforum* and other microorganisms, which begins with an interdigital lesion and induces lameness without joint involvement, with a good prognosis that includes grade 1. It can evolve into a deep interdigital lesion with necrosis and loss of skin integrity with lesions in the hooves and possible bone involvement, which may worsen the prognosis and, in some cases, generate deformities of the digits, grades 2 and 3.

It is known that in the region where this study was carried out, most farms did not implement preventive methods based on vaccination programs and strategies and, when implemented, used antibiotics empirically at the herd level (21). These factors can contribute to high and severe occurrences of foot rot. To minimize this impact, the main means of control and prevention of foot diseases in sheep flocks are currently the practices of hoof trimming, foot baths, elimination of chronic cases, periodic inspection of animals introduced into the flock, use of paddocks free of *D. nodosus* (14 days without animal movement), as well as antibiotic therapy when indicated, followed by vaccination program (6, 13, 16, 37).

Based on the macroscopic classification into degrees, it is possible to assist in understanding the severity of lesions observed in the field and future management measures and treatments that can be adopted. According to some studies, regular sheep hoof trimming practices correct abnormal growth and minimize the accumulation of organic matter in the area. This makes the hoof and interdigit less predisposed to dermatitis and contamination by *D. nodosus*. In addition, combined with topical treatment via foot bath, it facilitates the action of bactericidal substances on hooves that already present mild interdigital lesions (2, 18). However, an observational study carried out in England (13) and also observed by Prosser et al. (38) provide evidence that routine hoof trimming should be done carefully as it may be associated with a higher prevalence of lameness when hooves are trimmed and bleed, i.e., when damage to living tissue occurs, directly or through increased susceptibility to pathogens, that causes lameness, rather than the hoof trimming itself.

In the most severe lesions of grade 2, there is already a more pronounced inflammatory process with bone lesions. However, the prognosis is only favorable if these sheep are kept isolated in infirmary paddocks and without contact with the other animals in the flock, as there may already be highly virulent strains installed in the lesion. For this degree, treatment should be individual and with antibiotic therapy or bactericidal solutions in footbaths which is the most effective and viable way to combat the disease (16, 39–41).

Monovalent or polyvalent vaccines are being developed in search of a single tool that helps control and eradicate the causes of lameness, such as footrot disease (37). The major challenge of vaccination is to determine whether the vaccination strategy is appropriate for the herd. Immunity against *D. nodosus* is serogroup specific, with minimal or no cross-protection between serogroups. Similarly, some virulent strains belonging to different serogroups may be present in the same herd (42–44). An observational study demonstrated a reduced risk of lameness in flocks vaccinated with Footvax (45). The results showed that vaccination contributed to a small but significant reduction in the prevalence of lameness of approximately 20%. However, vaccination strategies are still most effective when combined with other methods. Rapid and individual treatment of lame sheep has been shown to be highly effective in reducing the lameness prevalence (38). The applicability of this rapid management when identifying sheep with sporadic lameness and knowing how to characterize the lesions early on, as described in grade 1 or even grade 2 with discrete/mild lesions, makes the treatment strategy effective and prevents the progression of the disease and worsening of the lesions. In addition, actions such as the use of antibiotic therapy, preventive and therapeutic footbaths, quarantine of newly introduced sheep in the flock and culling of sheep that present lesions compatible with grade 3 described in this work, reduce the risks of transmission and spread of the disease in the flock (18, 43, 46, 47).

## Conclusion

Ovine pododermatitis is a debilitating disease and a source of economic concern in most sheep producing countries. Through histopathological and radiographic studies, we could explain and grade the lesions observed clinically. Such lesions at bone level also can explain clinical observations in animals that heal from acute lesions but still have chronic bone damage. This complemented and deepened the clinical classification system based on the grade of lesions, will allow technicians and farmers to more consistently define treatment, control and prevention strategies that will be beneficial for animal health and, consequently, economically beneficial for sheep producers.

## Data Availability

The raw data supporting the conclusions of this article will be made available by the authors, without undue reservation.
