# Supplementary material for: Histopathological and radiographic characterization of the lesions of pododermatitis in sheep: support for the establishment of the foot injuries degree and its prognosis
Source: Front Vet Sci. 2025 Apr 25;12:1567665. doi: 10.3389/fvets.2025.1567665 (PMC12063351; doi:10.3389/fvets.2025.1567665)
Supplement: Supplementary file 1 [file Table_1.DOCX]

**Supplementary Materials**

**Table 1** | Main epidemiological and macroscopic aspects of cases of foot lesions observed in sheep from farms in the Southwest Rio Grande do Sul Mesoregion.

| **Farms** | **City** | **No. sheep on farms** | **No. sheep with lameness** | **Foot injury intensity by farms** | **Type of treatment** |
| --- | --- | --- | --- | --- | --- |
| 1 | Uruguaiana | 108 | 10 | III | TH + ATB |
| 2 | S. Livramento* | 350 | 100 | III | TH + FB |
| 3 | Uruguaiana | 300 | 10 | II | TH |
| 4 | Uruguaiana | 2500 | 250 | I, II, III | FB + ATB |
| 5 | Uruguaiana | 2000 | 400 | I, II, III | TH + FB + ATB |
| 6 | S. Livramento | 250 | 30 | III | TH + FB + ATB |
| 7 | Itaqui | 29 | 3 | III | FB |
| 8 | Itaqui | 120 | 12 | I, II | TH |
| 9 | Itaqui | 400 | 80 | I, II, III | TH + FB + ATB |
| 10 | Itaqui | 455 | 50 | I, II | TH + ATB |
| 11 | S. Livramento | 970 | 50 | I | TH + FB + ATB |
| 12 | S. Livramento | 4000 | 22 | III | TH + FB + ATB |
| 13 | S. Livramento | 1700 | 20 | II | TH |
| 14 | S. Livramento | 1200 | 120 | III | TH |
| 15 | Uruguaiana | 170 | 20 | II | TH + FB + ATB |
| 16 | Uruguaiana | 880 | 352 | I, II, III | TH + FB + ATB |
| 17 | Maçambará | 400 | 40 | III | ATB |
| 18 | Alegrete | 78 | 3 | II | ATB |
| 19 | Alegrete | 600 | 30 | I, II, III | FB + ATB |
| 20 | Barra do Quaraí | 280 | 20 | II | TH + FB |
| 21 | Uruguaiana | 200 | 20 | I, II, III | TH + ATB |

^*^Santana do Livramento. I = mild, II = moderate, III = severe; TH = trim hooves, FB = foot bath, ATB = antibiotic. Table adapted from Silveira CS, et al. 2016 (21).
